# Strengthen of magnetic anisotropy of Au/Co/Au nanostructure by surface plasmon resonance

**DOI:** 10.1038/s41598-019-45122-1

**Published:** 2019-06-14

**Authors:** Yusuke Kikuchi, Takuo Tanaka

**Affiliations:** 1 School of Materials and Chemical Technology, Tokyo Institute of Technology, 2-12-1 Ookayama, Meguroku, Tokyo 152-8550 Japan; 2 Metamaterials Laboratory, RIKEN Cluster for Pioneering Research, 2-1 Hirosawa, Wako, Saitama 351-0198 Japan; 3 Innovative Photon Manipulation Research Team, RIKEN Center for Advanced Photonics, 2-1 Hirosawa, Wako, Saitama 351-0198 Japan

**Keywords:** Nanophotonics and plasmonics, Magnetic properties and materials, Metamaterials

## Abstract

We experimentally demonstrated the increase of in-plane magnetic anisotropy in Au/Co/Au nanostructures by localized surface plasmon resonance (LSPR). When an array of Au/Co/Au square patch nanostructures was illuminated with linearly polarized light whose wavelength was 750 nm, the localized surface plasmons were resonantly excited in the nanostructures. From the measurement results of polar magneto-optical Kerr effect curves, we observed the magnetic anisotropy field increase in the Au/Co/Au nanostructure due to the excited surface plasmons. The in-plane magnetic anisotropy energy density was increased about 24%.

## Introduction

Magnetic properties of magnetic devices strongly depend on their materials, the fabrication process, and the surrounding environments. When we want to modulate the magnetization properties of these devices after they have been made, external stimuli such as heat, pressure, and so on, are applied^1–3^. To develop ultra-fast magnetic memory devices, several magnetization control techniques have been proposed, including the voltage-driven magnetization switching^4–6^, photo-induced magnetization^7–9^, and helicity-dependent magnetic switching^10–12^. However, these phenomena are only observed under the limited conditions like using ultrathin magnetic film with a thickness below 1 nm^4–6^, in ultra-low temperature^7–9^, or using a femtosecond pulsed laser irradiation^10–12^. Recently, several groups have reported that the magneto-optical effect is enhanced at the resonant wavelength of the localized surface plasmons (LSPs) in metal nanostructures at room temperature^13–17^. This research field is now called as “magneto-plasmonics^18–20^”. Our group also has reported that the longitudinal magneto-optical Kerr effect and magneto-optical figure of merit in Au/Co/Au nanostructure is enhanced by the localized surface plasmons excitation^21^. According to these reports, the LSPs affect (and modulate) on the magneto-optical property of the magnetic thin film, but it still remains undiscussed whether the surface plasmon excitation affects the magnetization mechanism or not.

Magnetic anisotropy is an essential factor for practical applications, because it determines the thermal stability of magnetic devices, like magnetic random access memory (MRAM) and magnetic recording devices (e.g. hard disk drive), just to name a few^22,23^. In these devices, the magnetic anisotropy usually decreases when the temperature of the material increases. Heat-assisted magnetic recording (HAMR) technology exploits this phenomenon by modulating the magnetic anisotropy through temporary heating of the recording media and then writing tiny (high-density) data bits on the media^24,25^. In this paper, we report the first demonstration of the increase of the in-plane magnetic anisotropy in Au/Co/Au nanostructure under the LSPR and we also discussed its mechanism.

## Methods

**Figure 1:**
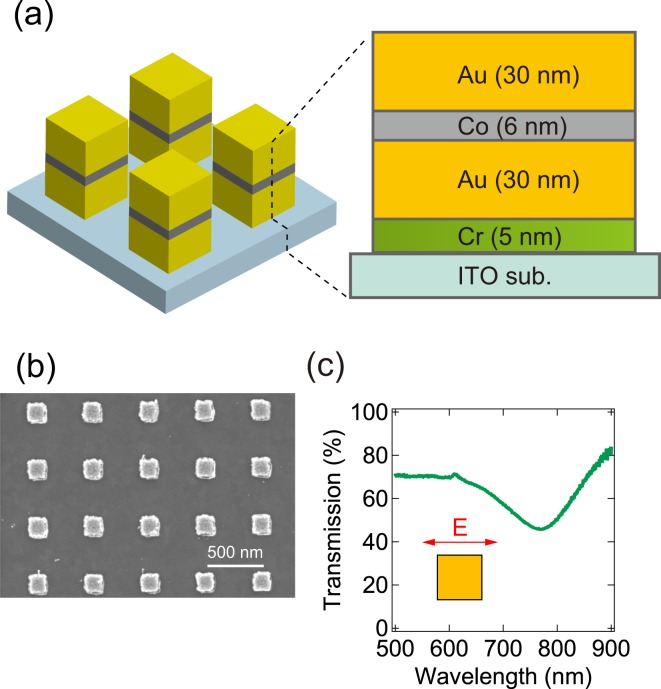
(**a**) Schematic of the fabricated nanostructure and the stacking structure of the thin film (cross-section view). (**b**) SEM image of fabricated Au/Co/Au nanostructures. (**c**) Transmission spectra of Au/Co/Au nanostructure.

Figure 1(a) shows the schematic of the fabricated nanostructure including a cross section of the thin film’s stacking order. An array of these square patch nanostructures (170 nm × 170 nm in lateral direction) consisting of Cr (5 nm)/Au (30 nm)/Co (6 nm)/Au (30 nm) were fabricated on an ITO substrate using electron beam lithography. The Cr layer serving as an adhesive layer for Au, was initially deposited by thermal evaporation. The succeeding Au, Co, and again Au layers were continuously deposited using electron beam evaporation right after thermal evaporation in the same chamber and the same vacuum condition. Finally, after the liftoff process, Au/Co/Au nano square patch array was obtained. Figure 1(b) is the scanning electron microscope (SEM) image of the fabricated nanostructures. The distance between the center of each square in the *x* and *y* directions is 480 nm. The optical properties of the sample were characterized by measuring transmission spectra using a UV-visible spectrometer (Ocean Optics, USB2000 + XR1-ES). The characteristics of the sample structure’s magnetic anisotropy at room temperature were evaluated by the magnetic field dependence of the longitudinal and the polar magneto-optical Kerr effects since the magneto-optical Kerr effect reflects the magnetization of materials^26^. The in-plane magnetization curve was evaluated by a longitudinal magneto-optical Kerr effect (L-MOKE) measurement system based on a polarization modulation technique using a photoelastic modulator (PEM)^26,27^. In this experiment, diode lasers with wavelengths of v640 and 785 nm were used as light sources and these laser beams were introduced onto the sample surface at a fixed incident angle of 14° and their polarization were set parallel to the edge of the nanostructure surface. The incident laser intensity was set to 142 mW/mm^2^. The magnetic field of 1000 Gauss was applied parallel to the sample surface.

The out-of-plane magnetization curve was evaluated via a polar magneto-optical Kerr effect (P-MOKE) measurement system (MODEL BH-M800UV-HD-10, NEOARK CORP.). In this system, a xenon lamp was used as a light source, and the wavelength (*λ*) of the incident light was selected by a monochromator. The plane of incidence was set parallel to the edge of the nanostructure surface. The incident angle was fixed at about 5°. The incident light intensity in P-MOKE measurement is estimated to be less than 1.4 μW/mm^2^. A magnetic field of ±20 k Gauss was applied perpendicular to the sample plane.

## Results

First of all, in order to evaluate the plasmonic properties of the fabricated Au/Co/Au nanostructure, we measured the transmission spectra as shown in Fig. 1(c). The polarization direction of the incident light was parallel to the edge of nano square patch as the inset of Fig. (c). From this result, we confirmed that the absorption peak originating from the SPR was observed only at *λ* = 770 nm, and no other absorption peaks were found in the range of *λ* = 500 to 640 nm. This result indicates that LSPs excitation can be switched on and off by changing the wavelength of the incident light and this is why we chose light sources whose wavelength are close to 770 nm in the L-MOKE and P-MOKE experiments to induce LSPR.

**Figure 2:**
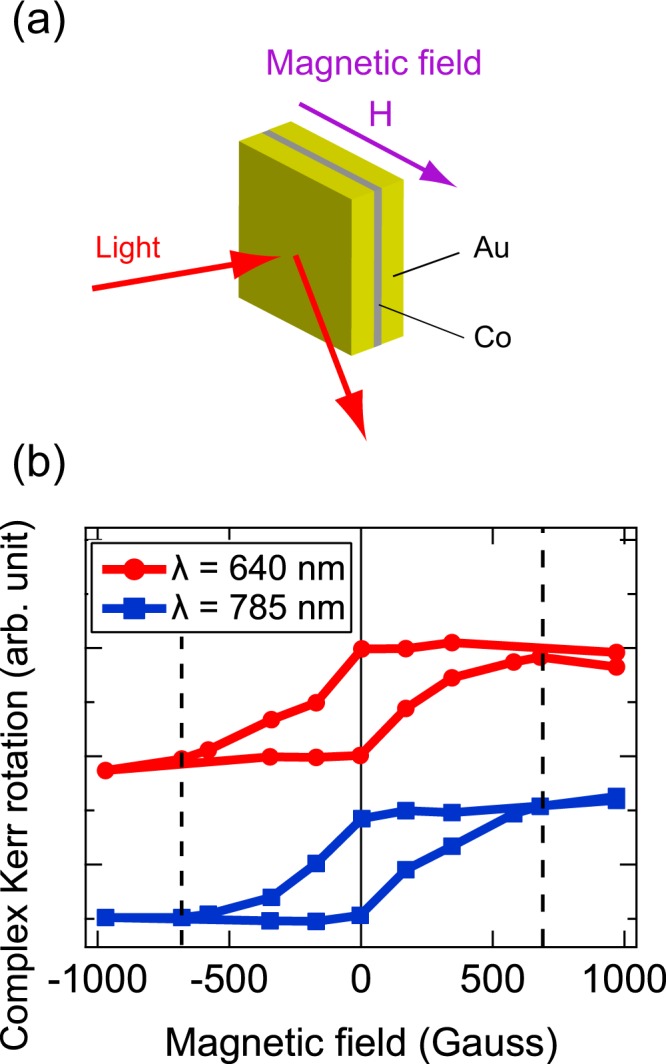
(**a**) Longitudinal magneto-optical Kerr effect (L-MOKE) measurement configuration. Magnetic field (H) is applied parallel to the surface of the Au/Co/Au nanostructure. (**b**) Magnetic field dependence of normalized complex Kerr rotation in longitudinal configuration (L-MOKE curve) for Au/Co/Au nanostructure at two different incident wavelengths (λ): 640 nm – off-resonance (red circles and line) and 785 nm – on-resonance (blue squares and line).

Figure 2 shows the relationship between the applied magnetic field and the complex longitudinal Kerr rotation (L-MOKE curve). Clear hysteresis loops of the L-MOKE curves were obtained for both *λ* = 640 nm (red circles, off-resonance) and 785 nm (blue squares, on-resonance). In addition, there is no significant difference of the saturation field even by changing the wavelength of the incident light.

**Figure 3:**
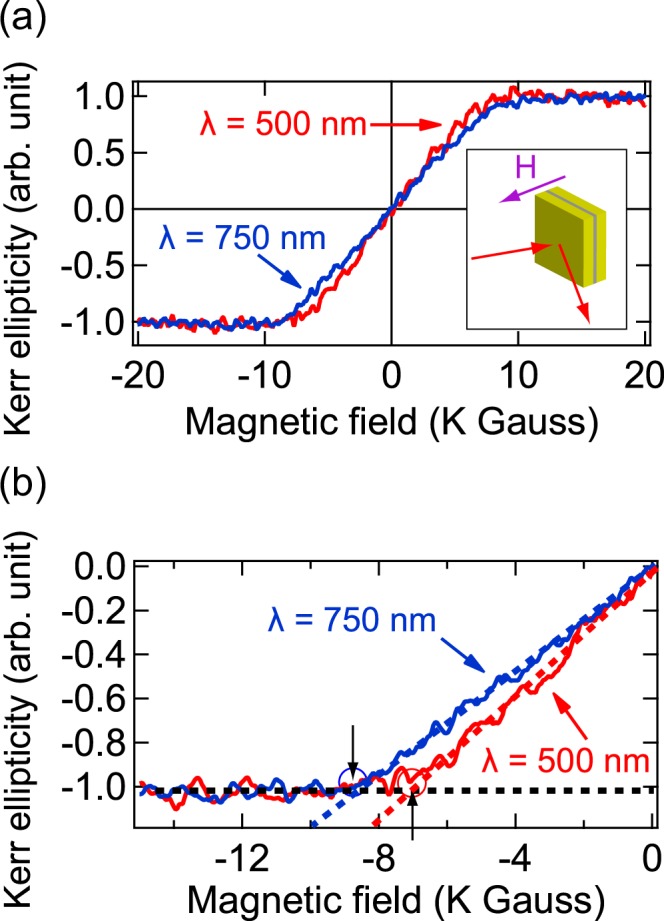
(**a**) Magnetic field dependence of normalized Kerr ellipticity in polar configuration (P-MOKE curve) for Au/Co/Au nanostructure at two different incident wavelengths (λ): 500 nm (red line) and 750 nm (blue line). The insert is schematic of polar magneto-optical Kerr effect (P-MOKE) measurement configuration. Magnetic field (H) is applied perpendicular to the surface of the Au/Co/Au nanostructure. (**b**) P-MOKE curves of Au/Co/Au nanostructure at the negative magnetic field region. Red dashed lines is the fitted data for 500 nm and blue dashed lines is for 750 nm. Black dashed line is the saturation of Kerr ellipticity. The intersection of the black dashed line and the red or the blue dashed lines gives the saturation magnetic field. Black arrows indicate the saturation magnetic fields for λ = 500 nm (red circle) and 750 nm (blue circle).

Figure 3 shows the relationship between the normalized Kerr ellipticity (*η*) and the external magnetic field in polar geometry (P-MOKE curve) measured with *λ* = 500 and 750 nm. As shown in Fig. 3(a), P-MOKE curves for both *λ* = 500 nm (red line, off-resonance) and 750 nm (blue line, on-resonance) do not exhibit any hysteresis loops unlike the L-MOKE curves. However, the saturated magnetization field, indicated by black arrows, in P-MOKE curves is about 10 times stronger than that of L-MOKE curves. These results imply that (1) Au/Co/Au nanostructure has a magnetic anisotropy and (2) the easy magnetization axis is parallel to the film plane. In addition, we found that there was a significant difference between the shape of P-MOKE curves for *λ* = 500 and 750 nm. To clarify this difference, we estimated the slope of the P-MOKE curve (*Δη*/*ΔH*) in the range from −6 k to 6 k Gauss for *λ* = 500 and 750 nm to be 0.148 and 0.120 (unit: arb. unit of Kerr ellipticity/k Gauss), respectively. We evaluated the magnetic anisotropy field (*H*_k_) in the P-MOKE curve. *H*_k_ is defined as the intersection of the fitted line of *Δη*/*ΔH* (*λ* = 500 nm, red dashed line; *λ* = 750 nm, blue dashed line) and the fitted line of the saturation Kerr ellipticity (black dashed line) in the range of −13 k to −20 k and 13 k to 20 k Gauss (Fig. 3(b)). The average magnitude of *H*_k_ in *λ* = 500 and 750 nm were about 7.10 k and 9.03 k Gauss, respectively. Reflection on some of these results made clear that *H*_k_ was drastically increased when the surface plasmons were excited in the structure (*λ* = 750 nm).

**Figure 4:**
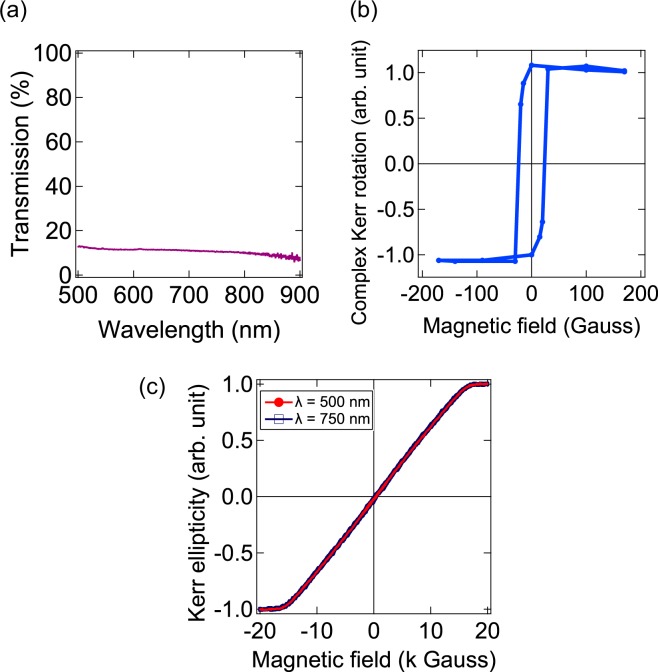
(**a**) Transmission spectrum of 20 nm-thick Co thin film. (**b**) Magnetic field dependence of normalized complex Kerr rotation in longitudinal configuration (L-MOKE curve) for 20 nm-thick Co thin film. (**c**) Magnetic field dependence of normalized Kerr ellipticity in polar configuration (P-MOKE curve) for the Co thin film with two different incident wavelengths (λ): 500 nm (red circles and line) and 750 nm (blue hollow squares and line).

In order to investigate the wavelength dependence of *H*_k_ in a Co thin film and compare with that of Au/Co/Au structure, we fabricated a bare 20 nm-thick Co thin film and measured its *H*_k_ at different wavelengths of irradiated light. The fabrication process of Co thin film is the same as that of the Au/Co/Au nanostructure. Figure 4(a) shows the transmittance spectrum of the Co thin film. No absorption peak was observed in the visible to near infrared region. This indicates LSPs were not excited in the bare Co thin film at the given wavelength region. Figure 4(b) shows the L-MOKE curve of the Co thin film for *λ* = 785 nm, and it presents clear hysteresis characteristics to the applied magnetic field. Figure 4(c) shows the P-MOKE curves of the Co thin film for *λ* = 500 nm and 750 nm. The overlapping of these two P-MOKE curves indicates that there is no wavelength dependence of *H*_k_ in the Co thin film. Therefore, we concluded that the remarkable increment of *H*_k_ in the Au/Co/Au nanostructure originated from the LSPs excited on Au.

## Discussion

From the magnetization curve, the in-plane magnetic anisotropy energy density (*K*_u_) in a thin film can be calculated as follows,

1 $${K}_{{\rm{u}}}=(\int {H}_{\perp }dM)-(\int {H}_{//}dM),$$

where *M* are magnetization and, *H*_⊥_ and *H*_//_ are magnetic field applied perpendicular and parallel to the surface of thin film, respectively. Using both L-MOKE and P-MOKE curves along with Eq. (1), we calculated the in-plane magnetic anisotropy energy density in the Au/Co/Au nanostructure with LSPs (*K*_u,wLSPs_) and without LSPs (*K*_u,w/oLSPs_), assuming a linear relationship between Kerr ellipticity (or complex Kerr rotation) and magnetization. The equations are as follows:

2 $${K}_{{\rm{u}},{\rm{wLSPs}}}=\frac{{M}_{s}}{{\eta }_{s}}{({\int }_{0}^{{\eta }_{s}}{H}_{\perp }d{\eta }_{k})}^{\lambda =750nm}-\frac{{M}_{s}}{{{\rm{\Phi }}}_{s}}{({\int }_{0}^{{{\rm{\Phi }}}_{s}}{H}_{//}d{{\rm{\Phi }}}_{k})}^{\lambda =785nm},$$

3 $${K}_{{\rm{u}},{\rm{w}}/{\rm{oLSPs}}}=\frac{{M}_{s}}{{\eta }_{s}}{({\int }_{0}^{{\eta }_{s}}{H}_{\perp }d{\eta }_{k})}^{\lambda =500nm}-\frac{{M}_{s}}{{{\rm{\Phi }}}_{s}}{({\int }_{0}^{{{\rm{\Phi }}}_{s}}{H}_{//}d{{\rm{\Phi }}}_{k})}^{\lambda =640nm},$$

where *M*_s_, *η*_s_, *η*_k_, Φ_s_, and Φ_k_ are saturated value of magnetization, saturated Kerr ellipticity, Kerr ellipticity, saturated complex Kerr rotation, and complex Kerr rotation, respectively. *M*_s_ = 900 emu/cm^3^ was used for 6 nm-thick Co thin film based on ref.^28^. Using Eqs (2) and (3), *K*_u,wLSPs_ and *K*_u,w/oLSPs_ were calculated as 0.383 × 10^6^ J/m^3^ and 0.310 × 10^6^ J/m^3^, respectively. This result implies that the in-plane magnetic anisotropy energy increases when LSPs are excited.

In a thin film, its magnetic anisotropy consists of both magnetocrystalline anisotropy and shape anisotropy^29,30^. In our case, the magnetic anisotropy energy modulation would come from the change of magnetocrystalline anisotropy because we used the same sample in all experiments and we verified that there was no sample deformation through all experiments.

To understand the results, we would like to discuss about the thermal effect on the magnetic anisotropy due to temperature increase from plasmon induced light absorption^31^. There are many theoretical and experimental works on the temperature dependence of magnetocrystalline anisotropy energy^32–34^. In all these works, decrease in the magnetocrystalline anisotropy energy has been observed with an increase of temperature. Our results, on the other hand, are different from these, because we observed that in-plane magnetocrystalline anisotropy energy increases as temperature increases. Here, we present possible scenarios to explain our results.

As stated above, the total in-plane magnetic anisotropy energy *K*_u_, which can be estimated from a magnetization loops shown in Figs 3 and 4(c), is written as a sum of shape anisotropy *K*_s_, which comes from magnetostatic property, and a magnetocrystalline anisotropy *K*_i_ as below,

4 $${K}_{{\rm{u}}}={K}_{{\rm{s}}}+{K}_{{\rm{i}}}.$$

In the case of thin film structure, whose thickness is thinner than its lateral dimension, the easy magnetization direction is parallel to the film surface and *K*_s_ has positive value. However, the size of structure becomes nanometer scale, the estimation and calculation of *K*_s_ is difficult and unreliable, because (1) the magnetization distribution in the nanostructure is complicated^35^ and (2) the magnetization of magnetic nanostructure cannot be measured directly. According to ref.^35^, magnetization vector in the square nanostructure are easily aligned to the in-plane direction and what we can say is *K*_s_ has positive value. Meanwhile, about *K*_i_, we can obtain its value neither by measuring nor calculation. Hence, it is possible to build up two hypotheses. One is *K*_i_ has negative value and another is it has positive value.

When we assumed that the sign of *K*_i_ is negative, which means the magnetization of nanostructure is perpendicular to the film surface, *K*_i_ reduces *K*_u_ and *K*_s_ should be larger than |*K*_i_| because *K*_u_ was positive which has already confirmed above using the L-MOKE and P-MOKE loops. Under this condition, when the temperature of the structure increases due to the plasmon resonance, *K*_i_ approaches zero and, as a result, *K*_u_ increases effectively. A similar phenomenon has been observed and reported in the magnetic thin film but never in nanostructures^36^.

Another possibility is the sign of *K*_i_ is positive, which is the same to that of Au/Co/Au thin film shown in Supplementary Information. When *K*_i_ has a positive value and decreases with the temperature increase by LSP excitation, *K*_u_ also always decreases. In this case, to explain our results showing an overall *K*_u_ increase by LSP resonance plausibly, it is necessary to introduce a new term *K*_p_, which is the plasmon-induced in-plane magnetic anisotropy energy. Moreover, even if the *K*_i_ decreases with increasing the temperature by LSP excitation, *K*_p_ increases *K*_u_ more than the decrease of *K*_i_. Finally, we propose a possible origin of plasmon-induced in-plane magnetic anisotropy. When LSPs are resonantly excited by light in metal nanostructure, collective electron oscillations are induced by the electric field of the incident light, which means that electric polarization is induced in the metal nanostructure by LSP excitation^37^. According to ref.^38^, by using a ferromagnetic insulator, they derived an expression to describe the electric polarization $(\overrightarrow{P})$ dependence of magnetocrystalline anisotropy energy (*K*_i_) as

5 $${K^{\prime} }_{{\rm{i}}}={K}_{{\rm{i}}}+{(\frac{\partial {K^{\prime} }_{{\rm{i}}}}{\partial \overrightarrow{P}})}_{0}\cdot \overrightarrow{P},$$

where ${K^{\prime} }_{{\rm{i}}}$ is the $\overrightarrow{P}$-dependent *K*_i_. Therefore, magnetocrystalline anisotropy energy increase via LSP resonance-induced electric polarization may be essential for the overall *K*_u_ increase by LSP resonance.

In summary, the increase of magnetic anisotropy energy in Au/Co/Au nanostructure was initially observed experimentally under the localized surface plasmon resonance. The increase of in-plane magnetic anisotropy energy density was estimated to be 0.73 × 10^5^ J/m^3^. We suggest that the increase of the magnetic anisotropy energy originates from the alteration of magnetocrystalline anisotropy by LSPs excitation.

## Supplementary information

Supplementary Information
