# Supplementary material for: Strengthen of magnetic anisotropy of Au/Co/Au nanostructure by surface plasmon resonance
Source: Sci Rep. 2019 Jun 14;9:8630. doi: 10.1038/s41598-019-45122-1 (PMC6570655; doi:10.1038/s41598-019-45122-1)
Supplement: Supplementary file 1 — Supplementary Information [file 41598_2019_45122_MOESM1_ESM.docx]

**Supplementary Information**

**Strengthen of magnetic anisotropy of Au/Co/Au nanostructure by surface plasmon resonance**

Yusuke Kikuchi^1,2^ and Takuo Tanaka^1,2,3,^

^1^ *School of Materials and Chemical Technology, Tokyo Institute of Technology, 2-12-1 Ookayama, Meguroku, Tokyo 152-8550, Japan*

^2^*Metamaterials Laboratory, RIKEN Cluster for Pioneering Research, 2-1 Hirosawa, Wako, Saitama 351-0198, Japan*

^3^*Innovative Photon Manipulation Research Team, RIKEN Center for Advanced Photonics, 2-1 Hirosawa, Wako, Saitama 351-0198, Japan*

**Electric field intensity distributions of Au/Co/Au nanostructure**

Here the calculation results of electric field intensity distributions in Au/Co/Au nanostructure are presented. Numerical calculations based on a finite element method were performed using a commercial software package (COMSOL Inc., COMSOL Multiphysics 5.3). In Au/Co/Au model used for the calculations, Au bottom layer (30 nm) was set onto an SiO_2_ layer with the size of 510×510×500 (height) nm^3^, Co layer (6 nm) was placed onto Au bottom layer, then Au top layer (30 nm) was set on Co layer. The size of the model comprising the Au/Co/Au nanostructure is 170×170 nm. Air was selected as a substance surrounding the Au/Co/Au. The x and y directions are parallel to each edges of Au/Co/Au model, z direction is perpendicular to the surface of the Au/Co/Au layer. A flat light source was set on the top of Au/Co/Au model that, generates the electromagnetic plane wave with polarization along the x-direction. Figure S1 shows the calculated electric field intensity (|*E*|) distributions of the Au/Co/Au nanostructures in x-z plane across the center of the Au/Co/Au nanostructure. The wavelength (*λ*) of the incident light was set to (1) 700 nm (localized surface plasmon (LSP) resonant wavelengths in Au/Co/Au model), and (2) 500 nm (off- LSP resonant condition). As shown in Fig. S1, under the LSP resonant condition, strong electric fields were generated not only at the air/Au interface but also on the surface of Co layer, while the enhancement and localization of the electric field did not appear at *λ* = 500 nm.


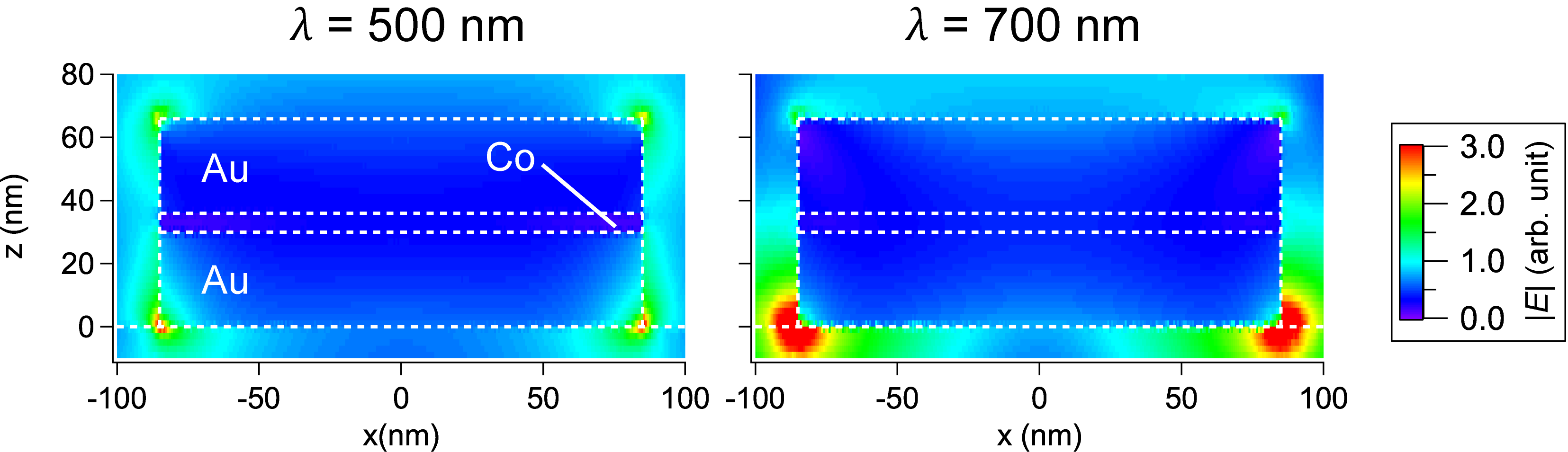


**Figure S1.** Simulated distributions of the electric field intensity (|*E*|) in the Au/Co/Au model in the x-z plane across the center of the Au/Co/Au model at the wavelength (*λ*) of 500 nm (off-LSP resonant condition) and 700 nm (on-LSP resonant condition).

**In-plane magnetic anisotropy energy in Au/Co/Au thin film**

Here in-plane magnetic anisotropy energy (*K*_u_) in Au/Co/Au thin film is presented. Thin films were prepared on an ITO-coated glass substrate. The stacking of the thin films is ITO-coated glass sub. / Cr (5 nm) / Au (30 nm) / Co (6 nm) / Au (30 nm). The fabrication method is same to that of the Au/Co/Au nanostructure. Figure S2 shows the L-MOKE (*λ* = 640 nm) and P-MOKE (*λ* = 750 nm) curves of the Au/Co/Au thin film. The saturated magnetic field of P-MOKE curve is about 110 times stronger than that of L-MOKE curves. This result indicates that (1) Au/Co/Au thin film has magnetic anisotropy and (2) the easy magnetization axis is parallel to the film plane.

*K*_u_ in the Au/Co/Au thin film was calculated using both L-MOKE and P-MOKE curves. The calculation method is same to that of the Au/Co/Au nanostructure. *K*_u_ was calculated as 5.41 × 10^5^ J / m^3^. As described in main text, *K*_u_ is written as a sum of shape anisotropy energy (*K*_s_) and magnetocrystalline anisotropy energy (*K*_i_). In the case of a thin film, whose lateral dimensions are treated as infinitely long compared to its thickness, shape anisotropy leads to an in-plane magnetization in thin film. Therefore the sign of *K*_u_ and *K*_s_ is same. Usually, *K*_s_ in a thin film is described by 2π*M*_s_^2^, where *M*_s_ is saturated magnetization in a magnetic thin film [S.1]. Moreover, *K*_i_ can be obtained by subtracting *K*_s_ from *K*_u_. Table S1 indicates *K*_u_, *K*_s_, and *K*_i_ in Au/Co/Au thin film. For calculating *K*_s_ in Au/Co/Au thin film, *M*_s_ = 900 emu / cm^3^ in 6-nm-thick Co thin film was used [S.2]. From the table S1, the sign of *K*_i_ is positive, which means that magnetocrystalline anisotropy leads to in-plane magnetization in Au/Co/Au thin film.

**Table S1.** *K*_u_, *K*_s_, and *K*_i_ in Au/Co/Au thin film.

| *K*_u_ (10^5^ J / m^3^) | *K*_s_ (10^5^ J / m^3^) | *K*_i_ (10^5^ J / m^3^) |
| --- | --- | --- |
| 5.41 | 5.09 | 0.32 |


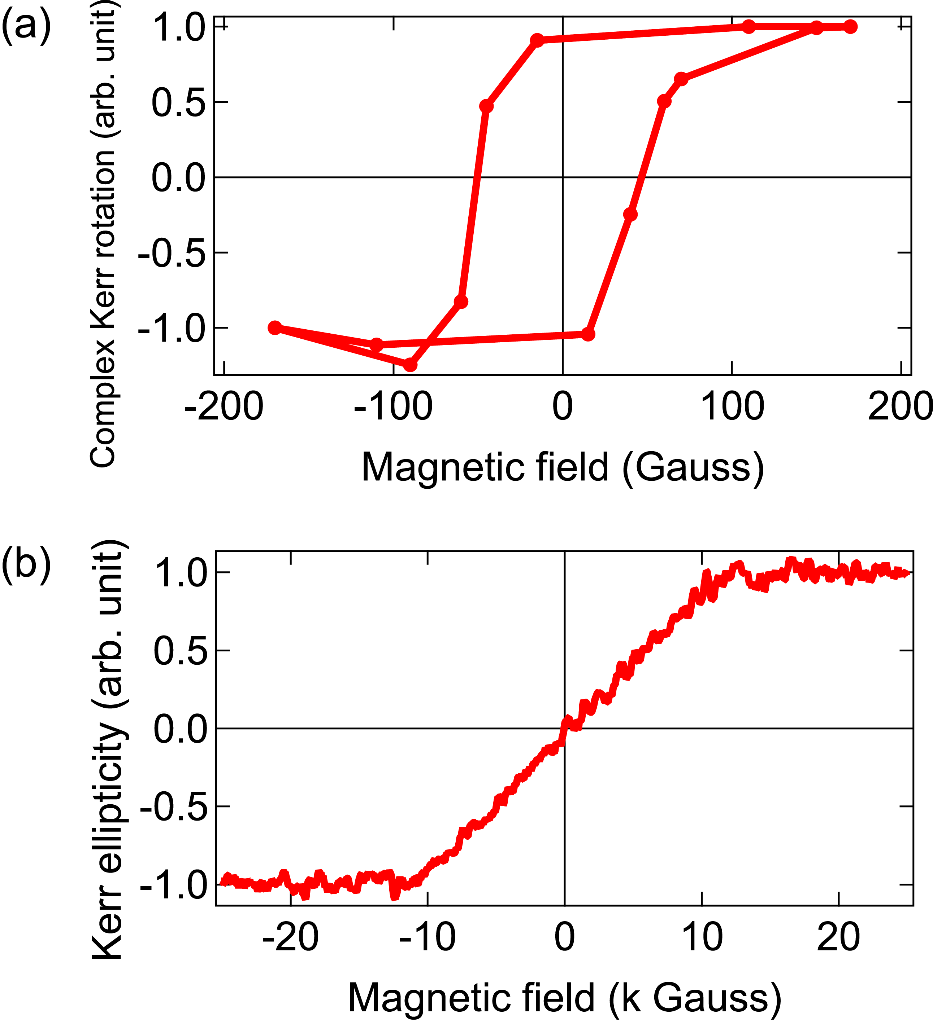


**Figure S2.** (a) L-MOKE, and (b) P-MOKE curve for Au/Co/Au thin film.

**Transmission spectra of Co nanostructure**

Here the transmission spectra of the Co nanostructure is presented. 20-nm-thick Co nanostructure was prepared on ITO-coated glass substrate. The dimension in lateral direction, lattice periodicity, and fabrication method are same to that of the Au/Co/Au nanostructure. Figure S3 shows the transmission spectra of the Co nanostructure. No clear absorption peak was observed in the visible to near infrared region. This result implies that the absorption peak observed at 770 nm in Au/Co/Au nanostructure was not associated with a diffraction.


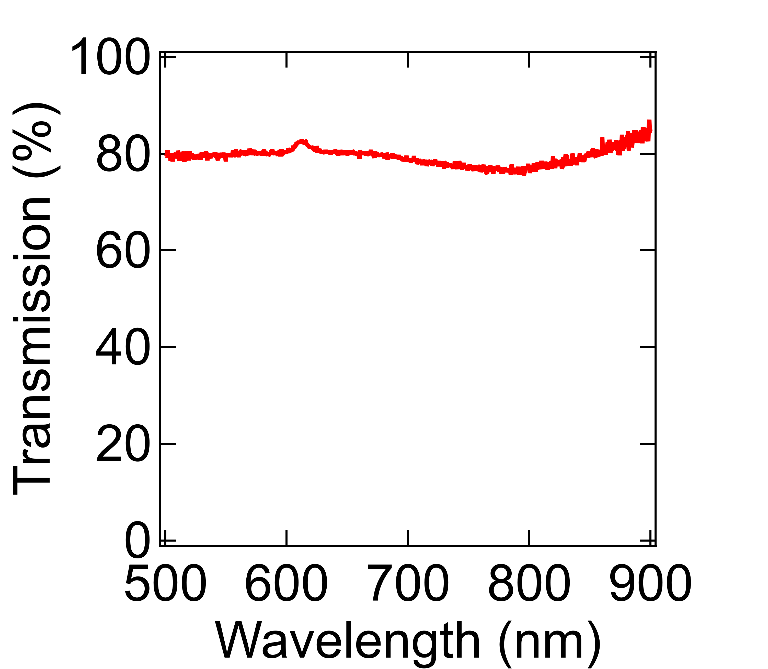


**Figure S3.** Transmission spectra of Co nanostructure.

**References**

1. T. Kojima, M. Mizuguchi, T. Koganezawa, K. Osaka, M. Kotsugi, and K. Takanashi, Japanese Journal of Applied Physics **51**, 010204-1 (2012).
2. T. C. Chuang, P. L. Su, P. H. Wu, and S. Y. Huang, Physical Review B **96**, 174406 (2017).
